# Probiotic Strains and Intervention Total Doses for Modulating Obesity-Related Microbiota Dysbiosis: A Systematic Review and Meta-analysis

**DOI:** 10.3390/nu12071921

**Published:** 2020-06-29

**Authors:** Ana López-Moreno, Antonio Suárez, Camila Avanzi, Mercedes Monteoliva-Sánchez, Margarita Aguilera

**Affiliations:** 1 Department of Microbiology, Faculty of Pharmacy, University of Granada, Campus of Cartuja, 18071 Granada, Spain; camiavanzi@hotmail.com.ar (C.A.); mmonteol@ugr.es (M.M.-S.); 2 Institute of Nutrition and Food Technology “José Mataix”, Center of Biomedical Research, University of Granada Armilla, 18016 Granada, Spain; asuarez@ugr.es; 3 IBS: Instituto de Investigación Biosanitaria ibs., 18012 Granada, Spain

**Keywords:** obesity, metabolic disorders, probiotics, strains, doses, intervention, obesogens

## Abstract

Obesity is a growing health threat worldwide. Administration of probiotics in obesity has also parallelly increased but without any protocolization. We conducted a systematic review exploring the administration pattern of probiotic strains and effective doses for obesity-related disorders according to their capacity of positively modulating key biomarkers and microbiota dysbiosis. Manuscripts targeting probiotic strains and doses administered for obesity-related disorders in clinical studies were sought. MEDLINE, Scopus, Web of Science, and Cochrane Library databases were searched using keywords during the last fifteen years up to April 2020. Two independent reviewers screened titles, abstracts, and then full-text papers against inclusion criteria according to PRISMA (Preferred Reporting Items for Systematic Reviews and Meta-Analyses) guidelines. From 549 interventional reports identified, we filtered 171 eligible studies, from which 24 full-text assays were used for calculating intervention total doses (ITD) of specific species and strains administered. Nine of these reports were excluded in the second-step because no specific data on gut microbiota modulation was found. Six clinical trials (CT) and 9 animal clinical studies were retained for analysis of complete outcome prioritized (body mass index (BMI), adiposity parameters, glucose, and plasma lipid biomarkers, and gut hormones). Lactobacillus spp. administered were double compared to *Bifidobacterium* spp.; *Lactobacillus* as single or multispecies formulations whereas most *Bifidobacteria* only through multispecies supplementations. Differential factors were estimated from obese populations’ vs. obesity-induced animals: ITD ratio of 2 × 106 CFU and patterns of administrations of 11.3 weeks to 5.5 weeks, respectively. Estimation of overall probiotics impact from selected CT was performed through a random-effects model to pool effect sizes. Comparisons showed a positive association between the probiotics group vs. placebo on the reduction of BMI, total cholesterol, leptin, and adiponectin. Moreover, negative estimation appeared for glucose (FPG) and CRP. While clinical trials including data for positive modulatory microbiota capacities suggested that high doses of common single and multispecies of *Lactobacillus* and *Bifidobacterium* ameliorated key obesity-related parameters, the major limitation was the high variability between studies and lack of standardized protocols. Efforts in solving this problem and searching for next-generation probiotics for obesity-related diseases would highly improve the rational use of probiotics.

## 1. Introduction

### 1.1. Probiotics for Dietary Supplementation Interventions

Probiotics remain a major complementary intervention resource for modulating microbiota dysbiosis, which is associated with several disorders and metabolic diseases [1,2].

Probiotics, according to the International Life Sciences Institute (ILSI), WHO, and the International Scientific Association of Probiotics and Prebiotics (ISAPP), are defined as “live microorganisms, which when administered in adequate amounts confer a health benefit on the host” [3]. Therefore, doses of certain probiotic strains might effectively modify misbalanced microbiota to make them healthier or, in other words, generate eubiosis [3,4].

Moreover, despite their numerous benefits, inadequate consumption of probiotics can even have undesirable effects, such as harmful metabolic activities, alterations of the integrity of the intestinal barrier, an inappropriate immune response, and the generation of antibiotic resistance genes and systemic infections [5,6]. Currently, the evaluation process of health claims determines the benefit of probiotics through clinical trials, which verify and demonstrate, in the last phase, the modulating capacities for the control of dysbiosis in patients with certain pathological disorders or phenotypes using omics complementary technologies [7]. It is also important to harmonize the knowledge on what specific probiotics should be recommended for dysbiosis, in what doses, and for how long.

Importantly, to obtain demonstrated benefits, appropriate probiotic doses must be administered under validated research intervention protocols. However, it is well known that an effective dose of probiotics might be influenced by a multitude of variables, including the final beneficial effect (objective), the specific probiotic strain used, and the vehicle and route of administration, which also have a decisive impact [8]. Most probiotic assays pay more attention to the final outcomes or modulated clinical parameters than to the establishment of a validated and harmonized protocol of specific doses and administration patterns [9]. Clinical studies or open science would allow clinicians and investigators to check trial outcomes and pursue interesting parallel uses of trial data, without compromising scientific integrity [10]. However, many probiotics trials do not show details regarding strains, doses, and patterns of administration. We believe that it is important to fill this gap since even in the long term, it would facilitate clinical and translational effects.

However, a personalized intervention plan with probiotics, prebiotics, or symbiotics should be applied to control the dysbiosis associated with obesity and metabolic disorders because of the complexity of each individual clinical case [11,12]. The selection of the right complementary treatment based on the probiotic formula is still far from being protocolized due to the discrete clinical improvements achieved as a consequence of the complexity of metabolic diseases [13] and the lack of integration data on probiotics, microbiota, and metabolites.

### 1.2. Microbiota Dysbiosis in Obesity-related Disorders: Obesogenic Substances

Microbiota is the microbial community that lives on and in the human body [14]. The composition of the microbiota may suffer variations due to several factors, including age, lifestyle, drugs, diet, antibiotics, and other environmental xenobiotics [15,16].

There exists demonstrated evidence of the association between gut microbiota and obesity in animals and humans [15,17,18]. However, the causal relationship and the underlying mechanisms remain unknown [19]. Importantly, recent data have highlighted the role of gut dysbiosis in the etiology and pathogenesis of metabolic disorders, including obesity, metabolic syndrome, type 2 diabetes mellitus, and non-alcoholic fatty liver disease [20]. Moreover, numerous animal studies and certain human studies suggest beneficial metabolic effects of microbial intestinal metabolites, such as short-chain fatty acids (SCFAs), which are vital for metabolic functions and the regulation of food intake and energy expenditure. Moreover, SCFA production from prebiotic consumption by supplemented probiotics might contribute to the reduction of obesity risk [21].

Furthermore, strong scientific evidence indicates that the gut microbiome not only reacts to diet but to other external contaminant substances, such as antibiotics and other xenobiotics, in ways that impact metabolic conditions, triggering obesity and other endocrine diseases, called obesogens [22]. They could also increase the energy harvest during obesity [16] or dysbiosis, referred to as an imbalance of the bacterial population in a natural colonization site, which can result in immune and metabolic diseases [17], diabetes mellitus type 2 [23], and/or metabolic syndrome. However, presently, very little is known regarding the molecular mechanisms underlying these triggered obesogenic effects [24]. Some groups of food xenobiotics or contaminants that have already been considered obesogens and modifiers of gut microbiota are endocrine disruptors [25]. However, few alternative strategies have been tested by now for counteracting, metabolizing, or neutralizing these xenobiotics and their body effects by appropriate dietary supplementation probiotics BPA [26]; Parahydroxybenzoate [27]; Phthalates [28,29]. In that direction, more research about bacteria components of the intestinal microbiota that could become new candidates for next-generation probiotics with specific biotherapeutic and detoxifying role could be developed [30]. Moreover, this area of research is now evolving in parallel with omics methodologies [7], improved identification, culturing, and next-generation sequencing technologies [31].

Taken together, the present review arises from the evidence of interrelated microbiota dysbiosis observed in obesity-related diseases and the possibility to prevent or palliate this misbalance with improved practical know-how of probiotic clinical interventions. To achieve this, we performed an extensive search strategy, review, extraction, and presentation of the most relevant and up-to-date scientific literature on effective probiotics. An overview of key specifications data from probiotic studies and their effective modulating role in gut microbiota dysbiosis in obesity were extracted, compared, and recommended according to better-obtained outcomes.

## 2. Materials and Methods

Methods used for this systematic review were started on April 2019 with reference to the Preferred Reporting Items for Systematic Reviews statement. The protocol and search strategy are shown in Table S1 following the requirement of the International Prospective Register of Systematic Reviews (PROSPERO).

### 2.1. Eligibility Criteria

All studies targeting probiotic strains and doses administered for obesity-related disorders in relation to data showing microbiota dysbiosis modulation capacities in humans or animal models were included. Two independent reviewers screened titles, abstracts, and then full-text papers against inclusion criteria according to PRISMA (Preferred Reporting Items for Systematic Reviews and Meta-Analyses) guidelines.

Specifically, to be included in the study, there were four mandatory inclusion criteria during abstract revision: (1) the study was published within the last fifteen years (i.e., between 2005 and 2020) and should specify (2) the probiotic strain used, (3) the dose, (4) the time/period of administration; and (5) criteria for screening full text according to the availability of specific data on its modulation microbiota capacity. Non-English-language manuscripts; documents containing no quantitative/obesity biomarker-specific data; studies with results on other kinds of dysbiosis or that were not concerned with diabetes, metabolic syndrome or obesity were excluded.

### 2.2. Search Strategy

**Figure 1:**
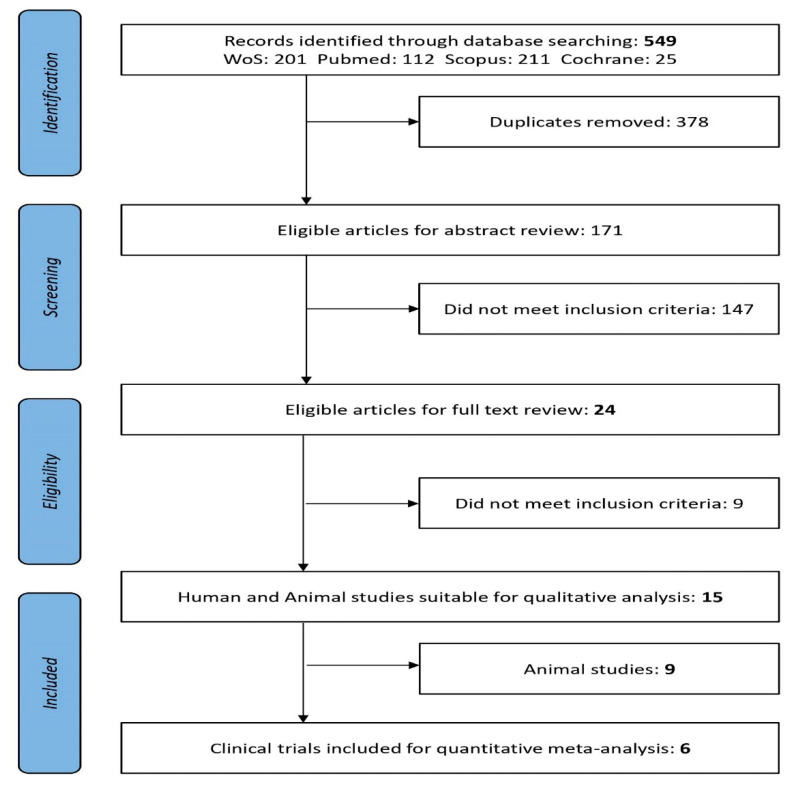
PRISMA flow diagram: Preferred Reporting Items for Systematic Reviews and Meta-Analyses [35].

Literature search and review of clinical studies were developed in collaboration with Granada librarian support using medical subject headings (MeSH) and the keywords (see below) under a stepwise procedure search and adapted to each database’s tutorials. The following electronic databases were searched from 2005 to 15 April 2020: MEDLINE/PubMed [32], Web of Science (Thomson Reuters Scientific), Scopus (Elsevier), and Cochrane Library [33]. Two independent reviewers (ALM and MA) revised titles and abstracts, then full-text publications with reference to the inclusion criteria. Study selection inter-rater agreement between the two reviewers was calculated as the proportion of positive agreement (PA) [34]. A PRISMA search on the topic of interest [35] flow diagram of the literature search summarizes the selection of the studies involved in the two main screening phases as previously explained (Figure 1).

Keywords: (Probiotic* AND microbiota AND obesity AND “endocrine disrupt*”); (Probiotic* AND microbiota AND obesity AND obesogens, xenobiotic*); (Probiotic* AND microbiota AND obesity AND hormon*); (Probiotic* AND microbiota AND obesity AND “drug metabol*”); (Probiotic* AND microbiota AND “metabolic syndrome” AND “endocrine disrupt*”); (Probiotic* AND microbiota AND “metabolic syndrome” AND xenobiotic*); (Probiotic* AND microbiota AND “metabolic syndrome” AND hormon*); (Probiotic* AND microbiota AND “metabolic syndrome” AND “drug metabol*”); (Probiotic* AND microbiota AND diabetes AND “endocrine disrupt*”); (Probiotic* AND microbiota AND diabetes AND xenobiotic*); (Probiotic* AND microbiota AND diabetes AND hormon*); and (Probiotic* AND microbiota AND diabetes AND “drug metabol*”) (Table S1).

### 2.3. Data Extraction and Analysis

Two independent reviewers (ALM and MA) extracted all relevant data in duplicate onto a Microsoft Excel spreadsheet. Publication authors were contacted if clarifications or specific data were missing. The following data were extracted for all studies meeting inclusion criteria: publication year, study design, number of participants, characteristics of the population, including the sample size (*n* = number of subjects) in the intervention group, gender, and age for animal and clinical trials; microorganism probiotic strains; dose; pattern of administration; modulation of the microbiota; modification of the clinical parameters, including (i) changes in body weight, BMI, waist circumference (WC), fat mass, fat percentage, and any alteration in parameters relating to weight; (ii) biomarkers related to metabolic changes: cholesterol levels (VLDL and LDL), triglycerides, glucose, insulin resistance or alterations in diabetes-related parameters; and (iii) hormone-related modifications.

The main data results from CT were qualitatively synthesized into Table 1. Comparative data extraction was done from animal studies having microbiota changes and relevant data were compiled (Table S2). Similar data studies but without microbiota were used for analyzing and calculating interventional total doses administered in obesity-related (Table S3). Moreover, further specifications and key results were retrieved from the overall analysis of clinical studies in order to visualize the relevance of the probiotics administered and the particular capacities in modulating microbiota and obesity-related dysbiosis.

Further data analysis of probiotic strains, effective doses, and pattern of administrations in obesity-related disorders were also retrieved from all collated data from clinical studies (15 selected studies and 9 overall analyzed reports that were none selected at the third step of the review because they did not fulfill microbiota modification criteria but contain primary and secondary relevant outcomes). Graphs depict preferent probiotic species, doses, and a pattern of administration in humans and animals and systematic narrative specifications are complementary for appropriate qualitative descriptions.

### 2.4. Risk of Bias (Quality) Assessment for the Selected Clinical Trials

Two independent reviewers (ALM and MA) assessed the risk of bias for each clinical trial selected using the Cochrane collaboration’s methodology [33]. In case of discrepancies, a third reviewer participated in this evaluation (AS). The risk of bias was tabulated for each study. Each item evaluated was classified as low risk (−; green cycle), high risk (+; red cycle), or unclear risk (?; yellow cycle), according to the quality recommendations described in Chapter 8 of the Cochrane Handbook of Systematic Reviews of Interventions [33]. This analysis and the corresponding figures were generated in RevMan 5.3 Review Manager (RevMan Computer program) Version 5.3. Copenhagen: The Nordic Cochrane Centre, the Cochrane Collaboration, 2019. Available at revman.cochrane.org.

### 2.5. Statistical Analysis

To calculate overall effect size for each study, the following steps were undertaken: (1) Baseline value in treatment group, baseline value in placebo group, endpoint in treatment group and endpoint value in placebo group were extracted. If baseline values were not reported in a study, only endpoint values were used; (2) Change ± SD from baseline was calculated for the treatment group and placebo group, separately; (3) Mean difference between changes from baseline in probiotics treatment group vs. changes from baseline in placebo group was calculated and used as overall effect size. When BMI, total cholesterol, LDL, HDL, TAG, glucose, CRP, leptin, adiponectin, and related parameters were reported in different units across the studies, alignment calculations or Hedges’s adjusted was used to calculate effect size. A random-effects model was used to pool calculated effect sizes. The I2–squared test to explore the heterogeneity, where an I2 > 75% is considered high heterogeneity and an I2 < 25% is considered low heterogeneity. Heterogeneity between subgroups was evaluated using a fixed effect model. Sensitivity analysis was performed by omitting one study at a time, to detect any significant changes in the results obtained. We used Begg’s rank correlation test and Egger’s regression asymmetry test to evaluate publication bias.

## 3. Results

The literature search focused on the selection of relevant data from probiotic studies, such as specific detailed microbial strains, doses, and patterns of administration during clinical interventions to effectively modulate dysbiotic microbiota in obesity and related endocrine and metabolic diseases.

### 3.1. Research and Scientific Evidence on the Probiotic Strains and Doses Administered for Obesity-Related Disorders

Obesity-related diseases are a worldwide concern and there is urgency to apply synergistic and multidisciplinary plans. Therefore, the initial overall search using the keywords “probiotics and obesity” showed an exponentially increasing trend of related studies carried out (Figure S1). Research and scientific evidence are summing data from different types of clinical studies especially during the last fifteen years. Consequently, after applying the specific searches and screening titles and abstracts, from 549 articles reviewed were eligible 171 for full-text revision (Figure 1). There was substantial PA between the reviewers of titles (PA = 0.78) and abstracts (PA = 0.84). Twenty-four articles met the inclusion criteria when Title and Abstract were revised; these were Clinical Trials (*n* = 6) and animal studies (*n* = 9). No papers reported for humans with xenobiotic obesogens (Figure 1). After applying the inclusion criteria, relevant outcomes from the studies fulfilling criteria and comparative data extraction from animal studies were presented in Table 1 and Table S2, respectively. 

The main extracted data from the selected studies were the following: sample (human or animal models), population size, probiotic strain/s, doses, administration pattern, individual microbiota modulation data, and common clinical and biochemical parameters related to weight, lipids and specific hormones related to weight gain, glucose, and lipid metabolisms (Table S1, Table S2, and Table S3).

**Figure 2:**
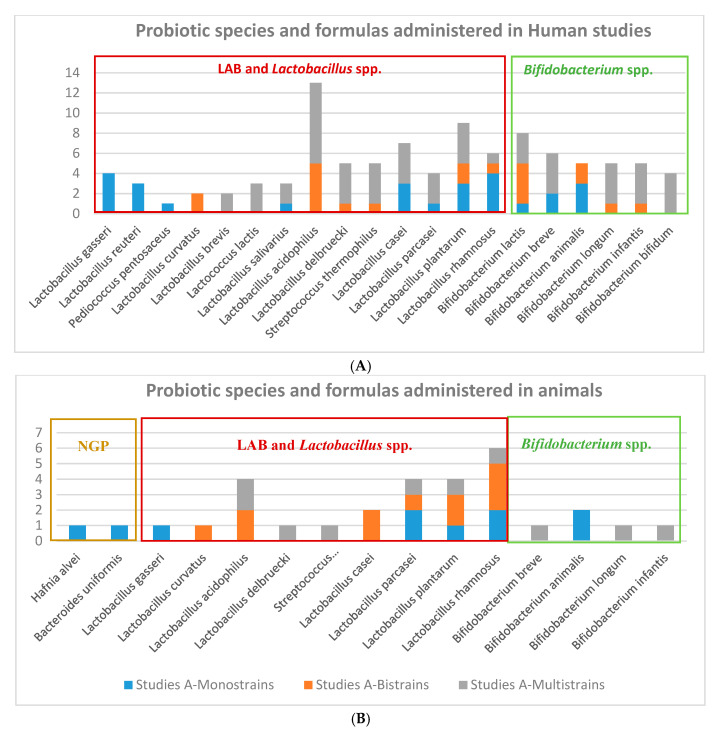
Probiotics formula administered in interventional obesity-related diseases in (**A**) humans (**B**) animal clinical studies.

**Figure 3:**
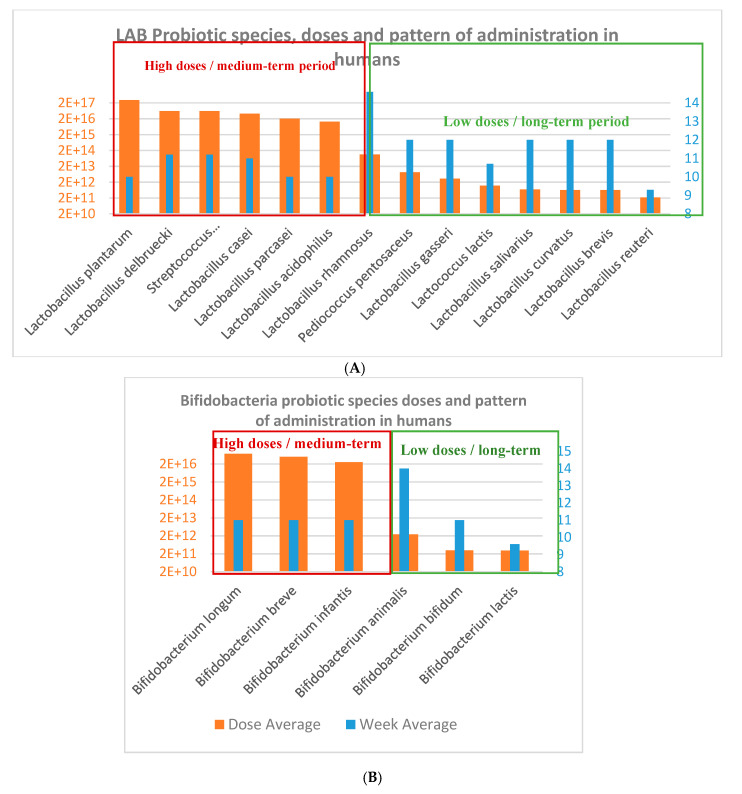
(**A**) *Lactobacillus* and Lactic Acid Bacteria (LAB) species and (**B**) *Bifidobacterium* species used in obesity-related disorders from human clinical trials.

**Figure 4:**
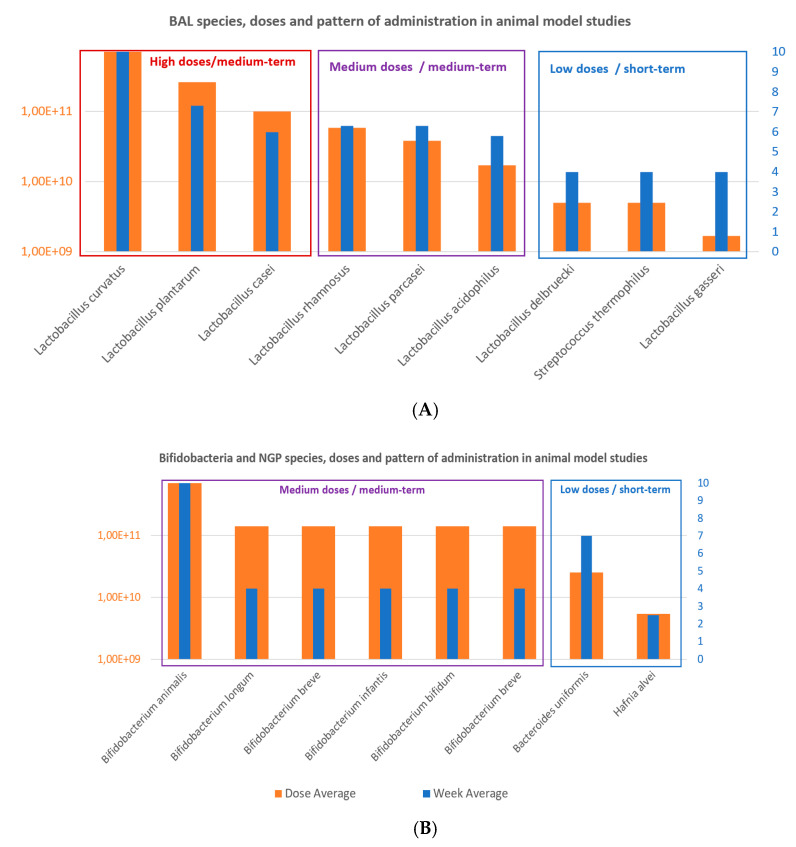
(**A**) *Lactobacillus* and LAB species and (**B**) *Bifidobacterium* and next-generation probiotics (NGP) species used in obesity-related disorders from selected animal clinical studies.

We found 24 reports resultant from the first-step selection focusing on probiotics species and strains used to effectively modulate parameters and biomarkers clinically relevant in obesity-related diseases. These documents were thoroughly analyzed for estimating the more common probiotic species used in obesity (Figure 2), calculating average doses (Figure 3) and pattern of administrations (Figure 4) to obtain comparative conclusions from human and animal clinical studies (Table 1 (6 CT), Table S2 (9 animal studies)). In order to obtain a more robust outcome, we included also data retrieved from nine reports containing a total of 47 suitable studies for the pursued analysis, summarized in the Table S3, 5 clinical studies [36,37,38,39,40] and 4 systematic reviews providing 12 studies from Cerdó et al. [41], 4 from Tenorio et al. [13], 7 from Sanz et al. [42], 20 studies from Koutnikova et al. [43]. None of these studies contained specific information on microbiota modulation capacities, so they could not be directly included for the main final microbiota analysis.

### 3.2. Probiotic Strains, Daily Doses and Intervention Total Doses in Obesity-related Clinical Studies

Probiotic strains: There is a trend of probiotics species and strains used in obesity-related human and animal clinical studies. There were more studies using a larger number of lactic acid bacteria (LAB) and specifically *Lactobacillus* species, comparing to *Bifidobacterium* species in humans and animal studies (Figure 4). It can be seen a higher arsenal of different probiotics species used for humans than for animals. Lactobacilli and Bifidobacteria formulations administered in human clinical trials were prepared mainly as monostrain (*Lactobacillus reuteri*, and *L. gasseri*), bistrains (*L. curvatus*), and multistrains (*L.acidophilus, L.brevis, L. salivarius, L. delbruecki, L. casei, L. plantarum, L.rhamnosus, L. paracasei*). However, Bifidobacteria strains administered to animals were mainly administered by multistrain preparation, in the VSL#3*commercial product mixture of 8 probiotics strains: *Lactobacillus acidophilus* DSM24735, *Lactobacillus plantarum* DSM24730, *Lactobacillus paracasei* DSM24733 and *Lactobacillus delbrueckii* subsp. *bulgaricus* DSM24734; *Streptococcus thermophilus* DSM2473; *Bifidobacterium breve* DSM24732, *Bifidobacterium longum* DSM24736, *Bifidobacterium infantis* DSM24737 [44]; or in a common multispecies preparation with 9 strains: *Bifidobacterium bifidum* W23, *Bifidobacterium lactis* W51, *Bifidobacterium lactis* W52, *Lactobacillus acidophilus* W37, *Lactobacillus brevis* W63, *Lactobacillus casei* W56, *Lactobacillus salivarius* W24, *Lactococcus lactis* W19, and *Lactococcus lactis* W58 [45].

**Figure 5:**
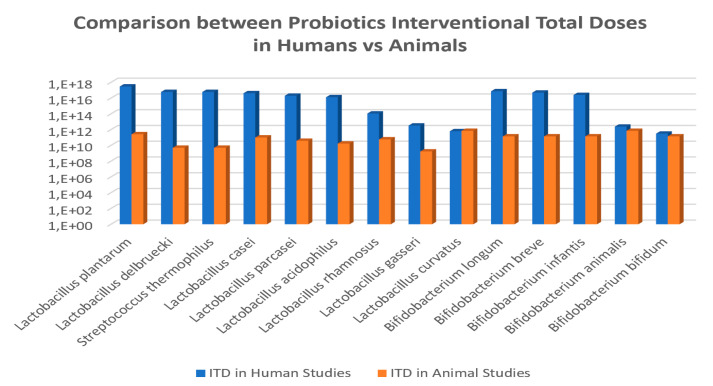
Comparison of interventional total doses (ITD) for common probiotic species administered to humans vs. animals in obesity-related disorders.

Probiotic doses and intervention total doses: In terms of the total intervention doses, the average and duration of probiotics intervention studies were 3.2 × 10^16^ CFU and 11.3 weeks in humans studies, which is equivalent to an average daily dose of 4 × 10^14^ CFU/day (Figure 4; Figure 5); oscillating between the maximum total dose of 3 × 10^17^ CFU and minimum dose of 2.2 × 10^11^ CFU for human CT. Additionally, the daily minimum and maximum doses varied 1 × 10^8^ CFU/day 1.35 × 10^15^ CFU/day, and the time of administration varied from 4 to 24 weeks.

**Figure 6:**
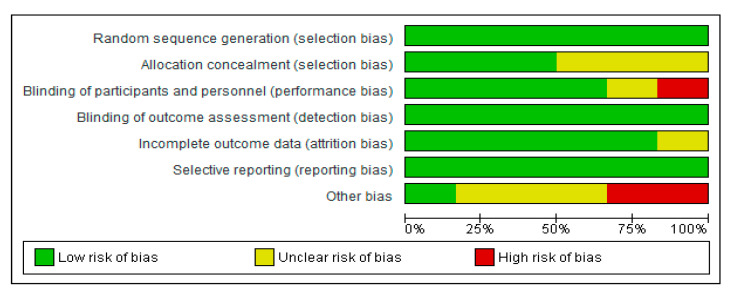
Risk of bias graph of CT: review authors’ judgments about each item as percentages across all included studies.

On the other hand, the average doses and duration of probiotics intervention studies in obesity-induced animal models were 1.5 × 10^11^ CFU and 5.5 weeks, which equivales to a daily dose of 4 × 10^9^ CFU/day (Figure 6); varying from maximum dose of 7 × 10^11^ CFU and minimum dose of 1.7 × 10^9^ CFU. Additionally, in the animal studies, the daily minimum and maximum doses varied from 2 × 10^6^ CFU/day to 1 × 10^10^ CFU/day, and the time of administration was also variable from 2.5 to 10 weeks.

None of the probiotic strain used in the obesity-related disorders triggered any safety concerns.

The analyses showed a trend that allow calculating a probiotics ITD extrapolation factor of 2 × 10^6^ CFU in average between human and animal clinical studies (Figure 5). The specific values for this ratio were of 1.2 × 10^7^ CFU for *Lactobacillus delbruecki* and *Streptococcus thermophilus*; 1.2 × 10^6^ CFU for *Lactobacillus plantarum*; 7.7 × 10^5^ for *Lactobacillus acidophilus*; 5.2 × 10^5^ for *Lactobacillus paracasei;* 4.2 × 10^5^ for *Lactobacillus casei*; 1.9 × 10^3^ for *L.rhamnosus*; 1.9 × 10^3^ for *L. gasseri*; 9 × 10^-1^ for *L. curvatus*; and for Bifidobacteria the ratio was of 5.3 × 10^5^ for *Bifidobacterium longum*; 3.6 × 10^5^ for *B. breve*; 1.8 × 10^5^ for *B.infantis*; 3.4 for *B. animalis*; and 2.2 for *B. bifidum*.

### 3.3. Probiotic Modulation Capacity on Individual Autochthony Microbiota and Clinical Parameters

#### 3.3.1. Impact of Probiotics on Individual Autochthony Microbiota

The studies analyzed showed more comprehensive data on the modulation of microbiota in animal studies (9) than in clinical trials (6), however they were not part of quantitative analysis because the extrapolation could not be done for comparisons between animals and humans.

In regard to human studies with impacting microbiota composition, no specific modulation pattern was shown within the selected probiotics supplementation CT (Table 1). Interestingly, three clinical trials did not register any change in the microbiota composition [46,47,48], which were all in line with a negative impact on BMI modifications. Conversely, [49] clinical trials performed in women showed microbiota modification capacities that were associated with positive effects on BMI.

In regard to probiotic formulas preferentially administered in human studies impacting microbiota composition (Table 1), the commercial multistrains VSL#3 product was used in two clinical trials with completely different administration patterns [46,50], both in terms of the doses (highest 10^15^ doses and normal dose 10^11^, respectively) and durations (18 and 6 weeks, respectively). Additionally, Jones et al. [46] did not find any changes in the composition of the microbiota after probiotics treatment, whereas Rajkumar et al. [50] showed an increase of total bacteria, specifically the total anaerobes.

We also found suitable results for 4 clinical trials with monostrain probiotic formula administered through different intervention total doses (ITD) from 8.4 × 10^9^ to 4 × 10^12^ CFU and heterogenous populations. Simon et al. [48] and Mobini et al. [47] administered two different strains of *Lactobacillus reuteri* to targeting different patient populations, and they found diverse results for the same clinical parameters. In the case of Mobini et al. [47], the patients had also diabetes type 2. Brahe et al. [51] studied the administration of one strain of *L. paracasei* and they did not find any changes in the clinical parameters of the patients (obese postmenopausal women). Finally, Sánchez et al. [49] administered a *Lactobacillus rhamnosus* strain and found significant differences between genders. While a decrease in the *Subdoligranulum* genus, coupled to weight loss and decreasing leptin levels were found in women, no significant differences were found in microbiota or any clinical biomarker in men treated.

Complementary results were obtained in regard to probiotics supplementation in obesity-induced animal studies (6 in mice, 2 in rats, and 1 in zebrafish) with impacting microbiota composition (Table S2). Monostrain formulas were successfully used: *Bifidobacterium animalis* subsp. lactis BB–12 [52] modulating the *Firmicutes*/*Bacteroides* ratio and decreasing glucose biomarkers and hormone related values but without changes in body weight; *Lactobacillus casei* CCFM419 [53] increasing *Bifidobacterium*, *Allobaculum*, *Bacteroidetes,* and *Lactobacillus* genera, SCFA-producing bacteria decreasing cholesterol biomarkers and hormone related values; *Lactobacillus rhamnosus* CNCM I-3690 [54] reducing *Bilophila wadsworthia* and increasing the presence of *Lactobacillus rhamnosus*, also decreasing glucose and insulin biomarkers but without changes in body weight; *Lactobacillus paracasei* subsp. *paracasei* (W8) [55] increasing *Lentisphaerae, Prevotella,* and *Lactobacillus* genera, also adiposity, energy intake and insulin levels were increased too; *Lactobacillus rhamnosus* BMI 501 [56] increasing *Rothia*, and allowing the appearance of genera such as *Mesorhizobium*, *Gordonia,* and Oxalobacteraceae family and decreasing biomarkers of obesity; Or bistrains well-known formula *Lactobacillus curvatus* HY7601 and *Lactobacillus plantarum* KY1032 [57] reducing bacteria diversity, weight, cholesterol and hormone biomarkers; *L. rhamnosus* LS8; *L. crustorum* MN047 [58] increasing the *Firmicutes*/*Bacteroides* ratio, decreasing body weight, and cholesterol levels and augmenting insulin tolerance.

Moreover, we found only two effective next generation probiotics in monostrain preparation belonging to *Bacteroides uniformis* [59,60] and *Hafnia alvei* HA4597 [61] for animal clinical studies exerting a positive clinical impact on the microbiota and obesity biomarkers.

#### 3.3.2. Impact on Obesity-related Clinical Parameters

The most significant modulation capacities of the clinical parameters were qualitatively extracted and shown in Table 1, choosing the variability of the three main clinical features linked to (i) weight parameters: BMI, waist circumference, fat, and/or adiposity; (ii) Biomarkers: plasma glucose, total cholesterol, TAG, LDL, VLDL, and liver glycogen levels; and (iii) hormone data levels: leptin, adiponectin, GLP-1, and Insulin indexes.

The studies were disaggregated if they presented multiple study groups with different results. This was the case of Mobini et al. [47] divided into Mobini a: low dose (group 1) and Mobini b: high dose (group 2); Sanchez et al. [49] divided into Sanchez a1: all subjects at 12 weeks, Sanchez a2: all subjects at 24 weeks, Sanchez b1: male at 12 weeks, Sanchez b2: male at 24 weeks, Sanchez c1: female at 12 weeks and Sanchez c2: female at 24 weeks.

**Figure 7:**
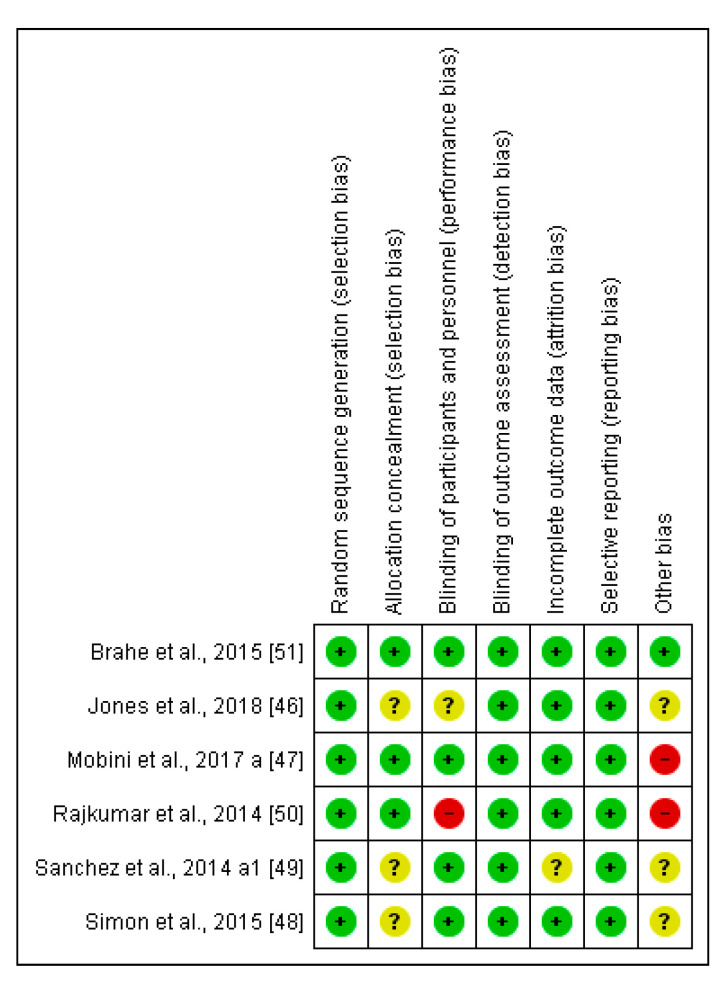
Risk of bias summary of CT: review authors’ judgments about each risk of bias item for each included study low risk (−: green cycle), high risk (+: red cycle), or unclear risk (?: yellow cycle).

The comprehensive method applied in selecting the final interventional documents and their outcomes guaranteed the quality of these clinical studies to obtain useful conclusions, both in animals and CT. Moreover, the possibilities in assessing the risk of biases of the 6 CT designs, execution, and outcomes increased the categorization of the applied quality standards. It gave added value to the evaluated CTs and allowed the reviewed results to be validated (Figure 6 and Figure 7).

**Figure 8:**
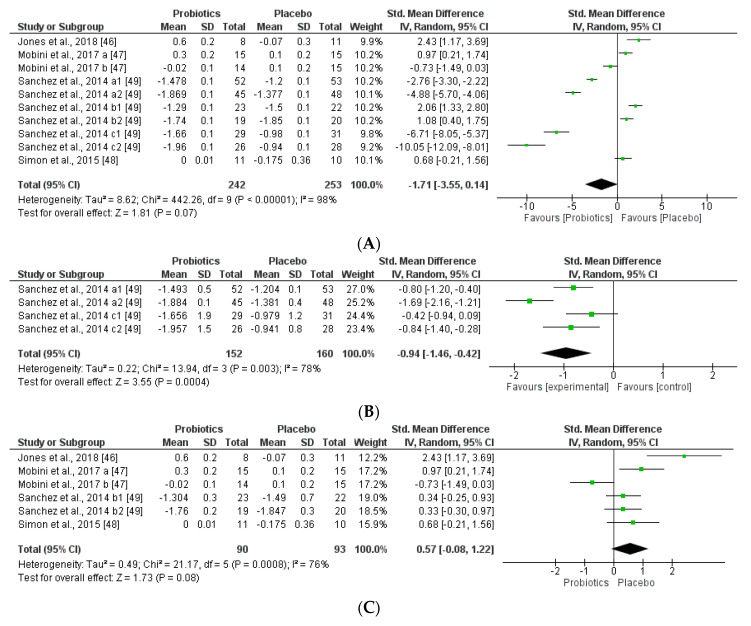
(**A**) Overall effect of probiotics on Body Mass Index (BMI) in selected Clinical Trials (CT). (**B**) Effect of probiotics on BMI in CT with impacting microbiota modifications. (**C**) Effect of probiotics on BMI in CT without impacting microbiota modifications.

**Figure 9:**
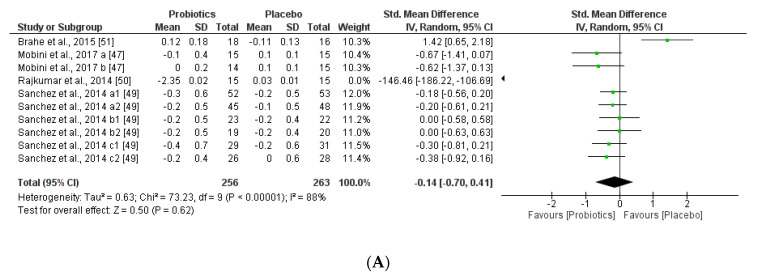

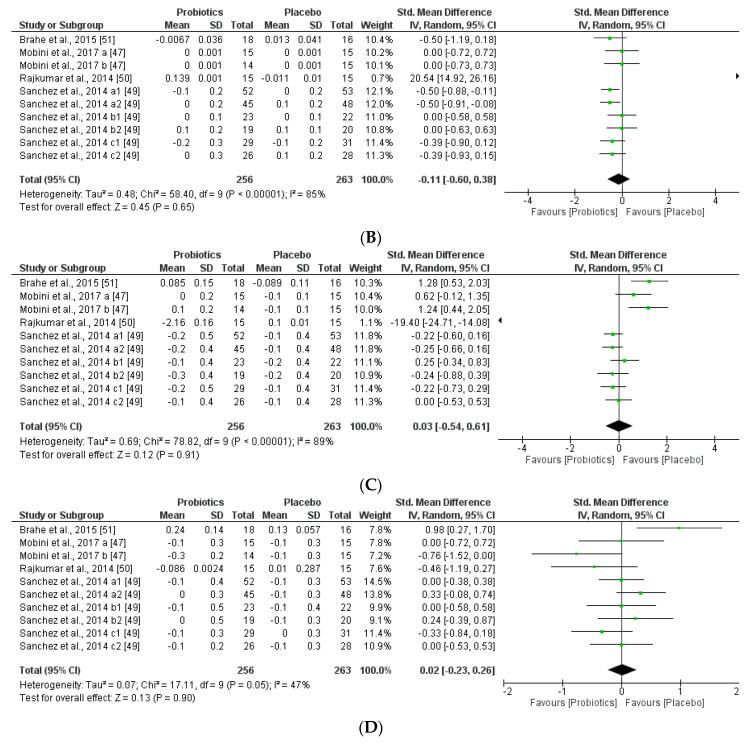
(**A**) Effect of probiotics on total cholesterol in CT. (**B**) Effect of probiotics on HDL-Cholesterol in CT. (**C**) Effect of probiotics on LDL-Cholesterol in clinical trials. (**D**) Effect of probiotics on TAG in clinical trials.

**Figure 10:**
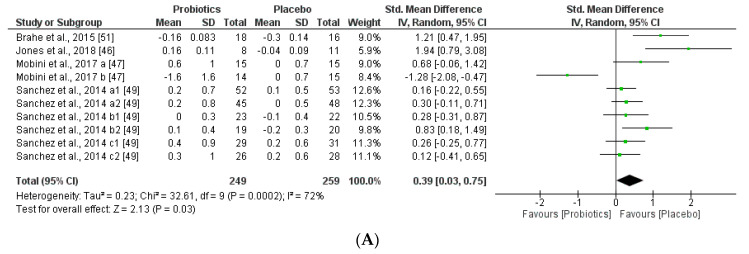

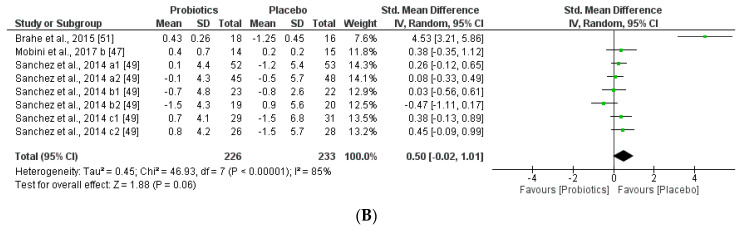
(**A**) Effect of probiotics on fasting plasma glucose in CT. (**B**) Effect of probiotics on CRP in CT.

**Figure 11:**
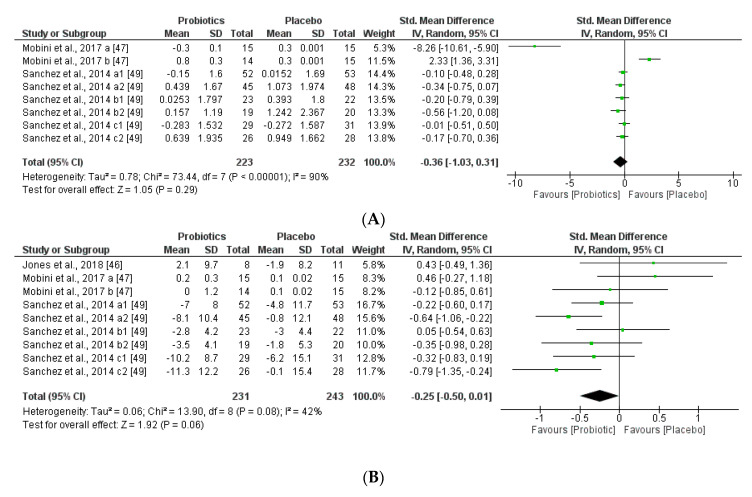
(**A**) Effect of probiotics on adiponectin in CT. (**B**) Effect of probiotics on leptin in CT.

We further reviewed, extracted, and highlighted the relevant information from the selected studies. Moreover, quantitative analysis through forest plot evaluations showed the statistical impact on each clinically relevant parameter and revealed the most significant changes and capacities of modulation of probiotics administered on BMI (Figure 8); Lipidic profile (Figure 9); Glucose and CRP levels (Figure 10) and Adiponectin and Leptin hormones (Figure 11).

Analyses through forest plots graphs showed the effects of probiotics in human studies in relation to BMI and microbiota modulation capacities and clinical parameter modifications were done. Black diamonds indicate the outcome for different probiotic formulas administered in each population studied.

The studies were disaggregated if they presented multiple study groups with different results. This was the case of Mobini et al. [47] divided into Mobini a: low dose (group 1) and Mobini b: high dose (group 2); Sanchez et al. [49] divided into Sanchez a1: all subjects at 12 weeks, Sanchez a2: all subjects at 24 weeks, Sanchez b1: male at 12 weeks, Sanchez b2: male at 24 weeks, Sanchez c1: female at 12 weeks and Sanchez c2: female at 24 weeks.

Interestingly, this meta-analysis showed that probiotics slightly improved lipid metabolism, specifically through modifying HLD-cholesterol levels and total cholesterol. While LDL-cholesterol and TAG levels seemed not to be modified by the probiotic supplements.

Remarkably, this meta-analysis showed that placebo group favored the glucose and CRP levels, which are correlated negatively with obesity-related symptoms and inflammatory responses.

The two obesity-related hormones, adiponectin (regulates glucose and lipid metabolisms) and leptin (regulates food intake and energy expenditure), may have a small but significant effect to decrease body weight and fat mass. This meta-analysis found that adiponectin and leptin concentrations were slightly decreased by supplementation with probiotics administered, however the trend of the outcome could not be correlated to glucose, lipid metabolism, but could be associated with the same tendency of the body weight (BMI) modifications by the probiotic supplementation groups. Heterogeneity percentages data were highly different for the two hormones, 90% for adiponectin and 42% for leptin.

## 4. Discussion

There is an exponential increase in the attention paid to the potential modulation of gut dysbiotic microbiota through dietary probiotics supplementation to prevent and/or improve metabolic diseases, such as obesity, diabetes, metabolic syndrome, and their comorbidities [62,63,64]. On the one hand, the use of the specific probiotic supplementation formulas was well substantiated through activity results in modifying key clinical biomarkers and safety demonstration [41,46,50]. On the other hand, discrete and sometimes unspecific outcome reported on the probiotics for obesity and especially scarce data focused on human microbiota modulation, make difficult to establish clear health assumptions and protocolization [65]. The beneficial effects of many probiotics, such as LAB, have been defended by its history of safe use [66], but currently scientific evidence demonstrating their benefits are available for many probiotics, such as the commercial multispecies VSL3 [67], and for monostrain e.g., *Lactobacillus casei* Shirota [43], *L. rhamnosus* GG [41], *Bifidobacterium breve B-3* [68]. However, most of these strains are administered through unharmonized pattern or clear effects due also to heterogeneous disorders treated [68]. Therefore, after identifying this redundant lack of information, the present work compiled the most commonly used probiotic strains, administration patterns, daily doses, and intervention total doses in human and animal studies (Figure 4, Figure 5, Figure 6 and Figure 7) in obesity-related disorders that showed modulation on key biomarkers. The majority of cited and collected probiotic strains were from genera *Lactobacillus* and *Bifidobacterium* giving heterogeneous clinical results. Several other species, such as *Saccharomyces boulardii* or *S. cerevisiae*, *Enterococcus faecium*, *Bacillus coagulans, B. clausii* commonly used in other GIT disorders, were not administered for these obesity-related studies. The choice of these probiotic species and strains to be administered in clinical studies seemed based on their previously proven beneficial effects, stronger activity or function, and safety aspects [69,70]. It was interesting to highlight the use in human studies of the most commonly combined probiotic formula, such as VSL#3 (*Streptococcus thermophilus, Bifidobacteria* (*B. breve, B. infantis, B. longum*)*, Lactobacillus acidophilus, L. plantarum, L. paracasei, and L. delbrueckii subsp. bulgaricus*), which has also been demonstrated to exert an impact in obesity, liver fat, steatosis, liver fibrosis, NAFL/NASH, NAFLD and other metabolic markers and gut hormones (GLP-1) [71,72]. We have no found definite clinical trials about the effect of VSL#3 on diabetes mellitus. However, the efficacy of VSL#3 on diabetes has been researched in obesity and non-obesity murine models [73]. Curiously, in two CTs [47,48], supplementation with different single strains of *L. reuteri* increased BMI but maintained the levels of lipid profiles and glucose, improving insulin sensitivity and insulin secretion in healthy and obese populations [74,75]. *L. paracasei ssp. paracasei F19* administration [51] maintained the stability of all clinical parameters, although some authors described a modification of *Ruminococcus torques* and *Eubacterium rectale*, and the latter is usually considered to belong to a group of beneficial bacteria [76]. As previously described, the *Lactobacillus* species are predominantly used, and they are consequently historically better described at strain level, such as *L. curvatus* HY7601 and *L. plantarum* KY1032 [77], which positively controlled all obesity parameters and slightly increase the *Firmicutes/Bacteroides* ratio. Moreover, species of different strains of *L. rhamnosus* [54,56] were administered, promoting modifications in microbiota, together with beneficial effects, such as in controlling inflammation [78] and lipid metabolism dietary parameters. However, other genera were also administered in obesity, such as *Bifidobacterium spp.* [79,80,81], *Streptococcus thermophilus*, and *Akkermansia muciniphila* [82,83].

One of the major hurdles for an accurate CT trial is to comprehend the operative dose of a probiotic at a strain-specific level. The beneficial role of putative probiotics is both strain-specific and dose-related or dose-dependent. It is expected that higher doses gave the most favorable (significant) biomarker-related metabolic effects with regard to e.g., adiposis, cholesterol, and triglyceride reduction. Our study has confirmed the differences in a strain-specific approach when selecting functional strains suitable for clinical studies. The importance of this issue has been emphasized in recent papers with regard to pre-clinical physiological studies on putative probiotic strains of LAB and *Bifidobacterium* administered.

In this review, we found significant differences in the probiotic doses of the interspecies assayed, where the minimum / maximum doses used in the CT, compared to the animal studies, ranged from 8.4 × 10^9^ CFU / 1.5–1,7 × 10^17^ CFU to 5.6 × 10^7^ CFU/ 7 × 10^11^ CFU, respectively. Despite this variation, at least one or several key clinical data (BMI, lipid parameters) were modulated, thus discretely improving the outcome pursued in the metabolic disease targeted subjects. In any case, the doses used in CT obesity-related patients were higher than the recommended nutraceutical formulas, which usually contains between 10^9^ and 10^10^ CFU/g or CFU/ml per day [9,84]. Different results were obtained through long-term vs. short-term oral supplementation of probiotics that seem also to exert differential effects on diversity and community structure of microbiota. In other studies, authors showed that short-term and long-term clinical studies alter the diversity and community structure of intestinal microbiota in mice with different physiological effects [85]. Long-term oral of *L. casei*sy13 enhanced the ability of colonization in the intestinal tract, however, a single time of oral dose had a greater effect on gut microbiota structure at phylum and genus. The impact in obesity patients should be different from the needs of stably colonizing the intestinal tract of the host. Therefore, doses establishment for *Lactobacillus* spp. and *Bifidobacterium* spp. requires extended validation methods, culturing [86] or molecular methods [87] to evidence the correlation of probiotics-microbiota-BMI modifications [88]; however, it remains controversial whether detection of this species is associated with the microbiota modification [89].

Although BMI is an indicator of the amount of body fat, it does not differentiate adiposity types, function, or metabolic implications [90]. Interestingly, we observed a correlation between the modification of the BMI and the microbiota. In cases where the microbiota is modified by probiotics, they obtain a decrease in BMI, however, in studies where the administered probiotic does not modify the microbiota, it produces an increase in BMI. In addition, we have observed differences between changes in BMI and the dose of probiotics administered the weeks of treatment and the gender of the study group.

In the present study, lipid metabolism parameters and concretely, triglyceride levels were not modified, while total cholesterol serum levels were slightly improved for the probiotics group (Figure 9). Other extensive probiotics studies have been reported to affect serum lipid levels in humans [91]. The same altered tendency profile of glucose (FPG) and CRP was observed through the meta-analysis, being favored for the placebo group. Therefore, the probiotics group seemed not to affect these metabolic and inflammatory interconnected biomarkers (Figure 9). Other studies showed that abnormal glucose hemostasis was associated with altered serum CRP concentrations [92]. Moreover, an association between obesity, insulin resistance, and inflammation needs prospective studies to better understand the mechanisms of the mediation of these relations by obesity. Several hypotheses showed that the effects of probiotics on metabolic endotoxemia and chronic inflammation seem to be accompanied by obesity [93]. Moreover, low-grade inflammation is also an important factor in the pathogenesis of diabetes, dyslipidemia, and comorbidities. Interestingly, other studies showed a positive correlation between BMI and CRP levels in obese patients. Moreover, CRP can be stimulated by leptin levels and CRP seemed to bind leptin receptor exerting modulations in both adipose tissue physiology as well as pathogenesis of obesity-related diseases [94]. Furthermore, leptin and adiponectin levels obtained through our meta-analysis were lower in the probiotic group than the placebo group. Furthermore, we could see a relationship between adiponectin and BMI, as in the case of Mobini et al. [47], whereas in Mobini a (low-dose probiotics) a decrease in adiponectin levels and an increase in BMI is observed, however in Mobini b (high-dose probiotics) they obtained higher levels of adiponectin and a reduction in BMI. In contrast to the other adipokines, circulating concentrations of adiponectin concentrations decrease in obesity and diabetes, as weight reduction increases plasma adiponectin concentrations [95]. Probiotics also change leptin levels, more specifically the duration of the treatment and the gender such as in Sánchez et al., not only the group of females had better results, but all groups during the longer treatment obtained better results.

Finally, we highlighted and claim the importance of including data related to the microbiota modulation capacities exerted by administered probiotics, together with clinical parameters and obesity modulation outcomes. Our searching strategy and extraction of data on microbiota were conducted on clinical trials and animal studies, in order to compare more available experimental data [96,97] that could improve the progress of the microbiome-obesity research field. Importantly, there was certain heterogeneity within the methodologies used to analyze changes in the microbiota (Table 1). Most researchers used metagenomic technologies for determining bacterial diversity focusing on V4 region of the 16S rRNA [46,47], V5—V6 region [48] and V123 and V456 region [49] through similar technologies, Illumina Miseq [46,47,48], SOLiD 5500xl [51] and 454 FLX [49] sequencers, except for one study that determined bacterial diversity through culturing methods [50]. This is in line with the results of several authors, who revealed that there still exist several technical and methodological limitations, which, together with the non-harmonized advances in microbiome-targeted interventions, make obesity prevention and standardized treatment through probiotic supplements more difficult [19,98]. Moreover, probiotic modulation capacities and the influence of gut microbiota status on the risk of obesity and intervention management had been better studied in animal models, in which experimental premises cannot be directly extrapolated to intervention in humans. Therefore, studies in humans still necessitate further robust and extensive investigations [99]. Furthermore, some innovative clinical interventions have shown that the modulation of the intestinal microbiota through fecal microbiota transplant (FMT) is clinically viable. Similarly, specific consortia of microbiota probiotics administration may also reduce several negative effects of metabolic diseases [100,101]. We also tried, to some extent, to envisage the available data on probiotics used to palliate the microbiota dysbiotic effects, such as the reduction of beneficial bacteria linked to the cumulative exposure to xenobiotic substances categorized as obesogens [102,103]. However, there were no direct clinical trials when analyzing the combined term strategy with xenobiotics. The absence of probiotics, metabolic diseases, and xenobiotic obesogens studies may indicate a new area of probiotic research for the future. Similarly, toximicrobiomics seem to be an emerging field of research. It integrates data on microbiology, bioinformatics, and toxicology, expanding the scope of personalized medicine [104,105]. However, we consider it important to highlight that current human exposure to xenobiotics is much more extensive through diet, food biotech, water, and pollution contaminants. Particular attention should be paid to obesogens and other manufactured products that are consumed daily [106]. Several authors focused on the underestimated exposure to xenobiotics, which could also be linked to microbiota dysbiosis, and its impact on obesity prevalence, which carries with it a high risk of cardiovascular disease, diabetes, and premature death worldwide [16]. Health concerns regarding the deleterious physiological effect of these obesogenic substances necessitate the characterization of the potential mechanisms of potential detoxifying probiotics [107,108,109,110].

In any case, all the selected probiotics showed marginal beneficial effects, which were extremely dependent on the administration patterns. Therefore, commercial probiotics for the future must be personalized according to the population group, specific microbiota dysbiosis, metabolic disorder to be treated, and the specific clinical status in order to limit all possible unwanted or unexpected effects. This systematic review suggests thorough microbiota analysis with complementary laboratory techniques (molecular methods; qPCR of specific taxa [111], culture methods [112]), beyond the common microbiota determination (V3—V4 16S RNA taxometagenomics), may be contributing to better determine variation in microbial populations; however, there are few data relating to these taxa determination.

Limitations of the current literature. First, the number of eligible studies was small and most chosen trials were performed in small sample size population; Second, analyzed studies used vastly different doses and strains of probiotics, and the selected trials were also heterogeneous in terms of disease states of populations, age, and other lifestyle factors. Further, usual dietary intakes were not checked in terms of possible probiotics consumption through the normal dietary patterns ç or concomitant treatments for obesity, diabetes type 2, metabolic syndrome that could also affect the gut microbiota composition and to be a cofounder of metagenomic studies; The current literature is also limited by methodological heterogeneity, in methods used for microbiota determination and the obesity-related biomarkers defining diagnostic, changes and evolution.; Moreover, there are documented controversies in obesity burden and prevalence of microbiota dysbiosis between obese, diabetes, and metabolic syndrome patients and even hormonal and/or pro-inflammatory disorders triggered by chemicals related to obesity [113,114]. And, some inconsistencies in metabolic syndrome data, reflecting the absence of internationally accepted definitions [13].

Future directions. To further our understanding of the preventive and improved role of probiotics in obesity-related disorders and to build upon a harmonized and standardize protocols or international guidelines for administration. Intensive and further research is required to investigate the effect of probiotics in human microbiota and how they relate to biomarkers levels modifications to cause improvements through high throughput methodologies. There is an urgent need to elucidate how probiotic administration and action would integrate the impact of multifactorial diseases according to precedent evaluations of the specific patient features, pathophysiological status, clinical and genetic factors, predispositions for developing metabolic diseases, and history of dietary xenobiotic obesogens exposure. Moreover, a joint effort to incentivize the publishing of accomplished probiotic clinical studies as open access (OA) literature [115] will make available more data for robust comparisons. New species of next-generation probiotics (NGP) [116] will constitute new standardized preventive and therapeutic tools for the near future.

## 5. Conclusions

The present systematic review has achieved and served to compile key data on the probiotic strains preferentially used for obesity-related metabolic disorders, effective doses, administration patterns, and the expected clinical benefits connected to microbiota dysbiosis modulation. Net beneficial trends could be interpreted from the meta-analysis, beyond the difficulties faced in aligning the heterogeneous data. Microbiota positive modulation capacity by probiotics seemed to be correlated with BMI and lipid biomarkers. However, due to the small number of studies that investigated the effects of probiotics in relation to the microbiota, it was unclear if they correlate with all the other glycemic, inflammation, and gut hormone parameters. Therefore, while higher doses of probiotic supplementations appear to be a useful adjuvant therapy for obesity-related patients comparing to other diseases, the role of the effective single species, e.g., *L. reuteri*, *L. rhamnosus,* and *L. paracasei* and multispecies probiotic formula (VSL3) would also require further investigation and efforts for standardization during intervention and determination of appropriate dose-dependent effects.

More standardization efforts and research intervention strategies should focus on modulatory microbiota capacities and envisage the development and use of next-generation probiotics whose formulation requires competent preclinical studies to show their efficacy and safety status. In overall terms, such advances and directions could help researchers, clinicians, dietitians, and nutritionists for using harmonized probiotics supplementary recommendations and targeted effects.

## Figures and Tables

**Table 1:** Summary of the probiotic strain’s effects in obesity and related metabolic disorders from selected clinical trials.

Report Reference	Population Characteristics	Probiotic Strain	Probiotic Doses CFU/day	Pattern Administration ITD (CFU)	Microbiota Modulation Capacities	Clinical Impact and Parameter Modifications		
						Weight	Biomarkers	Gut Hormones
Jones et al. [46]	19 Obese Latino adolescents	VSL#3* (Multispecies)	1.35 × 10^15^	16–18 Weeks 1.5–1.7 × 10^17^	No microbiota modification ↔ Firmicutes ↔ Bacteroides	↑BMI ↑Adiposity ↑Fat mass	↔ Glucose	↔ Leptin, GLP-1 and GLP-2 ↔ Insulin levels
Rajkumar et al. [50]	60 Overweight (30 women and 30 men)	VSL#3* (Multispecies) VSL#3*+ Omega-3	1.1 × 10^11^	6 weeks 4.6 × 10^12^	↑ Total bacteria ↑ Total anaerobes ↑ *Lactabacillus*, *Bifidobacteria*, *Streptococcus* and *Bacteroides.* ↓ Coliform, *E.coli*	↓ BMI	↑ HDL ↓ LDL, VLDL, TAG ↓ Glucose ↓hsCRP	↓ Insulin levels
Brahe et al. [51]	58 Obese postmenopausal women	*L. paracasei* *subsp.paracasei*F19	9.4 × 10^10^	6 weeks 4 × 10^12^	↑*Eubacterium rectale*and *Ruminococcus torques*	↔ BMI	↔ LDL, VLDL, TAG, Cholesterol, ↑hsCRP	↔ ISI↔ Leptin
Simon et al. [48]	10 Obese and 11 lean, all glucose-tolerant	*Lactobacillus reuteri* SD5865	2 × 10^10^	4 weeks 5.6 × 10^11^	No microbiota modification ↔ Total bacterial ↔Enterobacteria ↔Lactobacilli, ↑*Lactobacillus reuteri*	↑ BMI obese, ↔ Ectopic fat	↔ Glucose	↑ Insulin levels and C-peptide secretion ↑ GLP-1 and GLP-2
Mobini et al. [47]	46 (11 women and 36 men) Obese/diabetes	*Lactobacillus reuteri* DSM 17938	Group 1: 1 × 10^8^	12 weeks 8.4 × 10^9^	No microbiota modification Euryarcheota was initially elevated ↑Methanobacteria	↑ BMI ↑ Weight	↔ LDL, VLDL, TAG and Total Cholesterol	↔ Leptin levels.
			Group 2: 1 × 10^10^	8.4 × 10^11^	No microbiota modification	↔ BMI ↔ Weight	↔Lipid metabolism	↔ Leptin levels. ↑ ISI
Sánchez et al. [49]	125 Healthy overweight men and women	*Lactobacillus rhamnosus* CGMCC1.3724	3.2 × 10^8^	12 weeks 2.7 × 10^10^ 24 weeks 5.4 × 10^10^	↓*Subdoligranulum sp.* in women No microbiota modification in men	↓Weight in women ↔Weight in men	↔Total Cholesterol	↓Leptin levels in women.

* VSL#3 (Lactobacillus acidophilus DSM24735, Lactobacillus plantarum DSM24730, Lactobacillus paracasei DSM24733 and Lactobacillus delbrueckii subsp. bulgaricus DSM24734; Streptococcus thermophilus DSM2473; Bifidobacterium breve DSM24732, Bifidobacterium longum DSM24736, Bifidobacterium infantis DSM24737); ISI: Insulin sensibility Index; GLP-1: Glucagon-like peptide-1; GLP-2: Glucagon-like peptide-2; hsCRP: high sensitivity C-reactive protein; BMI: body mass index; aGLP-1: active Glucagon-like peptide-1; TAG: triglycerides; ALT: liver toxicity biomarker alanine transaminase; CFU: colony-forming unit; NAFLD: nonalcoholic fatty liver disease; LDL: low-density lipoprotein; HDL: high-density lipoprotein; VLDL: very low density lipoprotein. ↑Higher; ↓ Lower; ↔ Equal

## Supplementary Materials

The following are available online at https://www.mdpi.com/2072-6643/12/7/1921/s1, Figure S1: Overall searching with the key words “Probiotics and Obesity”; Table S1: Key words and search terms; Table S2: Summary of the probiotic strain’s effects in obesity and related metabolic disorders from selected animal model data studies; Table S3: Overall analysis of 10 reviews for retrieving data on Probiotic strains, doses and administration pattern in obesity-related clinical studies (CT /animal studies).

## Abbreviations

aGLP-1	Active Glucagon-like Peptide-1
ALT	Liver toxicity biomarker alanine transaminase
BMI	Body Mass Index
CFU	Colony Forming Units
CRP	C-Reactive Protein
CT	Clinical Trials
FMT	Fecal Microbiota Transplant
FPG	Fasting Plasma Glucose
GLP-1	Glucagon-like peptide-1
GLP-2	Glucagon-like peptide-2
HDL	High density lipoprotein
hsCRP	high sensitivity C-reactive protein
ISI	Index Sensitivity Insulin
LDL	Low density lipoprotein
NAFL	Non-Alcoholic Fatty Liver
NAFLD	Non-Alcoholic Fatty Liver Disease
NASH	Non-Alcoholic Steatohepatitis
NGP	Next-generation probiotics
OA	Open Access
PCR	Polymerase Chain Reaction
qPCR	quantitative Polymerase Chain Reaction
TAG	Triglycerides
VLDL	Very low-density lipoprotein
