# Supplementary material for: Probiotic Strains and Intervention Total Doses for Modulating Obesity-Related Microbiota Dysbiosis: A Systematic Review and Meta-analysis
Source: Nutrients. 2020 Jun 29;12(7):1921. doi: 10.3390/nu12071921 (PMC7400323; doi:10.3390/nu12071921)
Supplement: Supplementary file 1 [file nutrients-12-01921-s001.pdf]

SUPPLEMENTARY MATERIALS

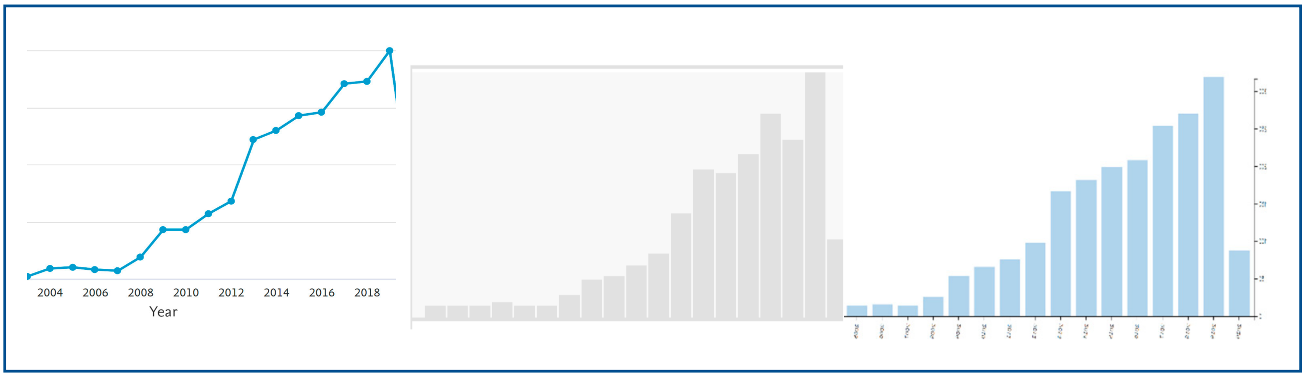

Supplementary material Fig.S1: Overall searching with the keywords “probiotics and obesity” (Images from Scopus, Pubmed, Web of Science, respectively)

PROTOCOL

Review question

Are there probiotics strains effective for modulating microbiota dysbiosis and clinical parameters of obesity-related diseases.

Searches

We are performing an overall analysis of clinical trials and animal models studies, published in English, conducted in adults and indexed in MEDLINE, Web of Science and Scopus databases. We are excluding any study on diabetic patients. The last day of publication to be included in this review is April 15, 2020. Table S1. Specific strings and search terms used for the review.

Supplementary Table 1S. Key words and search terms

String and Search terms	WoS (OA-NOA/T)	Pubmed (OA-NOA/T)	Scopus (OA-NOA/T)	Cochrane (SR / CT)
Probiotic* AND microbiota AND obesity AND “endocrine disrupt*”	0-0 / 0	1-0 / 1	0-2 / 2	0 / 0
Probiotic* and microbiota and obesity and xenobiotic*	8-7 / 15	4-6 / 10	4-11 / 15	0 / 0
Probiotic* and microbiota and obesity and hormon*	52-56 / 108	30 –19/ 49	41-48 / 89	0 / 12
Probiotic* and microbiota and obesity and “drug metabol*”	3-0 / 3	0-1 / 1	3-6 / 9	0 / 0
Probiotic* and microbiota and “metabolic syndrome” “endocrine disrupt*”	0-0 / 0	0-0 / 0	0-1 / 1	0 / 0
Probiotic* and microbiota and “metabolic syndrome” and xenobiotic*	2-1 / 3	1-1 / 2	2-1 / 3	0 / 0
Probiotic* and microbiota and “metabolic syndrome” and hormon*	10-17 / 27	2-2 / 4	13-9 / 22	0 / 3
Probiotic* and microbiota and “metabolic syndrome” and “drug metabol*”	0-0 / 0	0-0 / 0	2-1 / 3	0 / 0
Probiotic* and microbiota and diabetes and “endocrine disrupt*”	0-0 / 0	0-0 / 0	0-0 / 0	0 / 0
Probiotic* and microbiota and diabetes and xenobiotic*	4-2 / 6	3-3 / 6	3-4 / 7	0 / 0
Probiotic* and microbiota and diabetes and hormon*	20-15 / 35	18-18 / 36	25-30 / 55	0 / 10
Probiotic* and microbiota and diabetes and “drug metabol*”	2-2 / 4	2-1 / 3	1-4 / 5	0 / 0
Probiotic* and microbiota and infertility	0-0 / 0	1-0 / 1	0-2 / 2	0 / 0
Total reports extracted and analysed	101-100/ 201	62-51/113	94-119/213	0/25

Types of study to be included

Clinical trials, animal studies

Condition or domain being studied, Participants/population

Population subjects diagnosed (humans) or induced HFD (animals) with obesity-related diseases (Obesity, Metabolic Syndrome, Diabetes type 2)

Participants/population parameters analyses
Weight: BMI, body weight gain, adiposity
Biomarkers: lipid metabolism: cholesterol, LDL, VLDL, HDL, TAG, glucose (fasting plasma glucose FPG, resistance to glucose) and lipid metabolisms
hormone-related parameters: adiponectin, leptin, insulin sensitivity Index, incretin, GLP-1 and GLP2

Intervention(s), exposure(s)
We are including clinical studies where subjects were administered/exposed to probiotics. We are excluding exposures to products containing only prebiotics or symbiotics.

Comparator(s)/control
We are including clinical trials where subjects exposed to probiotics are compared to placebo.

Context

Main outcome(s)
Change of the dysbiotic microbiota components and changes of clinical parameters of the obesity-related disorders.

* Measures of effect: Variable

Additional outcome(s): Biochemical and hormonal parameters

Data extraction (selection and coding)
Abstracts of publications from PubMed, Web of Science, Scopus and Cochrane library databases were reviewed by ALM and MA. The list of the selected studies was reviewed by ALM, MA, and AS to eliminate any possible duplicated study and to resolve any discrepancies to select the final list of articles to be included.

Risk of bias (quality) assessment
ALM and MA independently assessed the risk of bias of the selected documents using the Cochrane collaboration´s methodology. In case of discrepancies, a third reviewer was involved in this evaluation (AS). Risk of bias was tabulated for each study and was classified as low, high and unclear as described in Chapter 8 of the Cochrane Handbook of Systematic Reviews of Interventions.

Strategy for data synthesis
We plan to provide a qualitative and narrative synthesis of the main results in the selected trials, organized by specific clinical, biochemical and gastrointestinal parameters. As a second level, we will organize results by the type of probiotic strain. Quantitative analyses were done using Forest Plots by RevMan 3.

Analysis of subgroups or subsets
If the necessary data is available, we will do subgroup analysis according to characteristics of the population or the probiotic strains administered.

Contact details for further information
Dr. Margarita Aguilera.
maguiler@ugr.es
Ms Ana López.
alopezm@ugr.es

Organisational affiliation of the review
University of Granada

Review team members and their organisational affiliations
Dr Margarita Aguilera. University of Granada
Ms Ana López. University of Granada

Type and method of review
Systematic review, qualitative and narrative synthesis for doses and pattern of administration, and meta-analysis or quantitative for CT

Supplementary Material Table S2. Summary of the probiotic strain's effects in obesity and related metabolic disorders from selected **animal model data studies**.

**Report Reference**	**Population characteristics**	**Probiotic strain**	**Probiotic Doses**	**Administration pattern Intervention total dose**	**Microbiota modulation capacities**	**Clinical impact and parameter modifications**		
						**Weight**	**Biomarkers**	**Hormones**
Park *et al.* [57]	36 Male C57BL / 6J **mice** Obesity by HFD	*Lactobacillus curvatus* HY7601 and *Lactobacillus plantarum* KY1032	**5 × 10^9** CFU/day each strain	**10 weeks** ***7 x 10^{11} CFU***	↓ Bacterial diversity ↓ *Verrucomicrobia* ↓ *Proteobacteria* ↑ Relative abundance 4*Firmicutes spp*	↓Body weight ↓ White adipose tissue	↓ ALT ↓ Cholesterol	↓ Plasma leptin and insulin ↑ Fatty acid oxidation gene expression: PGC1α, CPT1, CPT2, ACOX1.
Wang *et al.* [58]	72 male C57BL/6J **mice** Obesity by HFD	*L. rhamnosus* LS8; *L. crustorum* MN047	**1 × 10^9** CFU/day each strain	**10 weeks** ***7 x 10^{10} CFU***	↑ *Firmicutes*. ↓ *Bacteroidetes* and *Proteobacteria*	↓Body weight ↓epididymal fat	↓ LDL ↓ TAG	↑Insulin tolerance
Bomhof *et al.* [52]	80 Male Sprague-Dawley **rats** Obesity by HFD	*Bifidobacterium animalis* subsp. *lactis* BB-12	**1 × 10^{10}** CFU/day	**8 weeks** ***5,6 x 10^{11} CFU***	↓ Ratio *Firmicutes* / *Bacteroidetes*	↔ Body weight ↔ % Body fat	↓Glucose	↓ Insulin levels ↑insulin sensitivity ↑ GLP-2
Wang *et al.* [53]	male C57BL/6J **mice** Obesity by HFD and streptozotocin-induced	*Lactobacillus casei* CCFM419	**1 × 10^8** **1 × 10^9** **1 × 10^{10}** CFU/day	**12 weeks** ***8,4 x 10^9 CFU*** ***8,4 x 10^{10} CFU*** ***8,4 x 10^{11} CFU***	↑ *Bifidobacterium*, *Lactobacillus*, and SCFA-producing bacteria (10^8, 10^9 and 10^{10} CFU) ↑ *Allobaculum* (10^9 and 10^{10} CFU) ↑ *Bacteroides* (10^8 and 10^9 CFU) ↓ *Firmicutes* (10^9 CFU)	ND	↑Butyrate ↑Acetate ↔ Propionate ↓TAG and LDL	↑ GLP-1 (10^9 and 10^{10} CFU) ↓Insulin levels (10^8, 10^9 and 10^{10} CFU) ↑ Glucose tolerance

Natividad *et al.* [54]	Male C57BL/6J **mice** *Bilophila wadsworthia*<br>Obesity by HFD	*Lactobacillus rhamnosus* CNCM I-3690	**1×10^{9}** CFU/day	**5 weeks**<br>***$3,5 \times 10^{10}$ CFU***	↓ *Bilophila wadsworthia*<br>↑ *Lactobacillus rhamnosus*	↔ Body weight	↓Glucose<br>↑Butyrate<br>↑Propionate	↓Insulin levels
Cano *et al.* [39]	32 Male wild-type C57BL-6 mice<br>Obesity by HFD	*Bacteroides uniformis* CECT7771	**5×10^{8}** CFU/day	**7 weeks**<br>**$2,5 \times 10^{10}$ CFU**	↑ *Bacteroides, Bifidobacterium* and *Clostridium coccoides.*<br>↓ *Enterobacteriaceae.*	↓Body weight<br>↔ Adiposity	↓Cholesterol<br>↓TAG<br>↓ Glucose	↓Insulin levels<br>↓Leptin<br>↑ Glucose tolerance
Legrand *et al.*[61]	12 Male B6.V-Lep ob/ob JRj mice	*Hafnia alvei* HA4597	**3 × 10^{8}** CFU/day	**2,5 weeks**<br>***$5,4 \times 10^{9}$ CFU***	Low abundance of ClpB gene expressing *Enterobacterales* species	↓Body weight<br>↓Food intake	ND	ND
	22 Male C57Bl/6 JRj mice<br>Obesity by HFD		**4 × 10^{7}** CFU/day	**6,5 weeks**<br>***$1,8 \times 10^{9}$ CFU***	Negative correlation between BMI and genera of *Enterobacter, Klebsiella* and *Hafnia*	↓Body weight	ND<br>ND	
Lee *et al.* [55]	32 male Wistar **rats**<br>Obesity by HFD	*Lactobacillus paracasei subsp. paracasei* (*L. casei* W8®)	**4×10^{7}** CFU/g	**7 weeks**<br>***2×10^{9} CFU***	↑ *Lentisphaerae*<br>↑*Prevotella, L. paracasei* and *L. zeae.*	↑ Adiposity<br>↑ Energy intake	↑ Butyrate<br>↓ Propionate<br>↔ Acetate	↑ Insulin levels<br>↑ GLP-1 gene expression
Falcinelli *et al.* [56]	Juvenile **zebrafish** (*Danio rerio*)<br>Obesity by HFD	*Lactobacillus rhamnosus* BMI 501®	**2×10^{6}** CFU/day	**4 weeks**<br>***$5,6 \times 10^{7}$ CFU***	↑ *Rothia.* Appearance *Mesorhizobium, Gordonia* and Oxalobacteraceae.	↓Body weight	↓Cholesterol<br>↓TAG	↑ GLP-2 gene expression

Body weight gain; **ALT**: Liver toxicity biomarker alanine transaminase; **GLP-1**: Glucagon-like peptide-1; **GLP-2**: Glucagon-like peptide-2; **TAG**: Triglycerides; **LDL**: Low density lipoprotein; **HDL**: High density lipoprotein; **CFU**: Colony-forming unit; **NAFLD**: Nonalcoholic fatty liver disease; **HFD:** High fat diet.

Supplementary Material Table S3. **Overall analysis of 10 reviews for retrieving data on probiotic strains, doses and administration pattern in obesity-related clinical studies (CT /animal studies)**

Population characteristics			Probiotic Strains	Probiotic Doses	Administration Pattern Intervention Total Dose	Metabolic biomarkers
Alisi *et al.* [36]		44 Obese children with NAFLD	VSL#3*	9 x 10^{14} CFU/day	**16 weeks**<br>***1 x 10^{17} CFU***	↓ BMI.<br>↔ TAG and ALT.<br>↑GLP-1 and aGLP-1.
Cerdó *et al.* [41]	Osterberg *et al.* (2015)	20 Healthy non-obese male adults with HFD	VSL#3*	9 x 10^{14} CFU/ day	**4 weeks**<br>2,5 x 10^{16} CFU	↓ Weight and fat
	Vajro *et al.* (2011)	20 Obese children with hypertransaminasemia and ultrasonographic bright liver	*Lactobacillus rhamnosus* GG	1,2 x 10^{10} de CFU / day	**8 weeks**<br>*6,7 x 10^{11} CFU*	↓ BMI and visceral fat in combination with lifestyle interventions<br>↓ Hipertransaminasemia
	Kadooka *et al.* (2010)	87 Adults with obese tendencies	*Lactobacillus gasseri* SBT2055	1 x 10^{11} CFU / day	**12 weeks**<br>***8,4 x 10^{12} CFU***	↓ Weight body and BMI.
	Higashikawa *et al.* (2016)	62 Obese adults	*Pediococcus pentosaceus* LP28	1 x 10^{11} CFU/ day	**12 weeks**<br>***8,4 x 10^{12} CFU***	↓ BMI and waist circumference.
	Luoto *et al.* (2010)	113 Mother–child pairs for analysing the potencial overweight and obesity development	*Lactobacillus rhamnosus* GG	1 x 10^{10} CFU/day	**24 weeks**<br>***1,7 x 10^{12} CFU***	↓ Weight gain at 1 year of life and 4 years, no changes in later stages of development
	Minami *et al.* (2018)	80 Healthy pre-obese adults	*Bifidobacterium breve* B-3	2 x 10^{10} CFU/ day	**12 weeks**<br>***1,7 x 10^{12} CFU***	↓ Body fat mass
	Kim *et al.* (2018)	90 Obese adults (30 low-dose intervention and 30 high-dose intervention)	*Lactobacillus gasseri* BNR17	1 x 10^{9} CFU / day<br>1 x 10^{10} CFU / day	**12 weeks**<br>***8,4 x 10^{10} CFU***<br>***8,4 x 10^{11} CFU***	↓ Adipose tissue and waist circumference with high-dose intervention.
	Pedret *et al.* (2018)	126 Obese adults	*Bifidobacterium animalis* subsp. *Lactis* CECT 8145 or its heat-killed form	1 x 10^{10} CFU / day	**12 weeks**<br>***8,4 x 10^{11} CFU***	↓ BMI, waist circumference, no differences between live and heat-killed probiotic form

Cerdó *et al.* [41]	Famouri *et al.* (2017)	64 Obese children and adolescents with NAFLD	*Lactobacillus acidophilus* ATCC B3208, *Lactobacillus rhamnosus* DSMZ21690, *Bifidobacterium lactis* DSMZ 32,296 and *Bifidobacterium bifidum* ATCC SD6576	3×10^9 CFU/day<br>6×10^9 CFU/day<br>2×10^9 CFU/day and<br>2×10^9 CFU/day	**12 weeks**<br>***2,5 x 10^{11} CFU***<br>***5 x 10^{11} CFU***<br>***1,7 x 10^{11} CFU***<br>***1,7 x 10^{11} CFU***	↔ BMI and weight.<br>↓ Waist circumference.<br>↓ ALT and AST.<br>↓ Cholesterol, LDL and TAG
	Jung *et al.* (2015)	120 Obese adults	*Lactobacillus curvatus* HY7601 and *Lactobacillus plantarum* KY1032	5×10^9 CFU / day each strain	**12 weeks**<br>***4,2 x 10^{11} CFU***	↓ Weight body, waist circumference and fat.
	Sanchis-Chordá *et al.* (2018)	48 Obese children with insulin resistance	*Bifidobacterium pseudocatenulatum* CECT 7765	1 x 10^{9-10} CFU / day	**13 weeks**<br>***9 x 10$^{10-11}$CFU***	↓ Weight body
	Aller *et al.* (2011)	28 Patients with NAFLD	*Lactobacillus bulgaricus* and *Streptococcus thermophiles* (Nutricion Medica®)	5×10^8 CFU/day	**12 weeks**<br>***4,2 x 10^{10} CFU***	Improve liver function and glucose metabolism.
Koutnikova *et al.* [43]	Sharafedtino *et al.* (2013)	40 Obese patients with metabolic syndrome	*Lactobacillus plantarum* TENSIA	$7{,}5 \times 10^{12}$ CFU / day	**3 weeks**<br>***1,6 x 10^{14} CFU***	↓ BMI.<br>↓ Cholesterol.<br>↓ LDL and TAG.
	Takahashi *et al.* (2016)	137 Overweight patients	*Bifidobacterium animalis* ssp. *lactis* GCL2505	8×10^{10} CFU / day	**12 weeks**<br>***6,7 x 10^{12} CFU***	↓ Visceral fat area.
	Naito *et al.* (2017)	98 Obese patients	*Lactobacillus casei* strain Shirota	1 x 10^{11} CFU / day	**8 weeks**<br>***5,6 x 10^{12}CFU***	↓ LDL.
	Minami *et al.* (2015)	44 Obese patients	*Bifidobacterium breve* B-3	5×10^{10} CFU / day	**12 weeks**<br>***4,2 x 10^{12} CFU***	↓ Fat mass.
	Kadooka *et al.* (2013)	210 Obese Japanese adults	*Lactobacillus gasseri* SBT2055	$1{,}6 \times 10^{10}$ CFU / day	**12 weeks**<br>***3,4 x 10^{12} CFU***	↓ Abdominal visceral fat.<br>↓ BMI.
	Stenman *et al.* (2016)	225 Obese volunteers	*Bifidobacterium animalis* ssp. *lactis* 420	1 x 10^{10} CFU / day	**26 weeks**<br>***1,8 x 10^{12} CFU***	↔ Body fat mass.<br>↓ Waist circumference.
	Tripolt *et al.* (2013)	60 patients with metabolic syndrome	*Lactobacillus casei* Shirota	2×10^{10} CFU / day	**12 weeks**<br>***1,7 x 10^{12} CFU***	↓ BMI.

	Bernini *et al.* (2015)	51 Patients with metabolic syndrome	*Bifidobacterium lactis* HN019	2,7 x 10^{10} de CFU / day	**6 weeks**<br>***1,1 x 10^{12} CFU***	↓ BMI.<br>↓ Cholesterol, LDL and TAG.
Koutnikova *et al.* [43]	Ahn *et al.* (2015)	120 Overweight subjects	*Lactobacillus curvatus* HY7601 and *Lactobacillus plantarum* KY1032	1 x 10^{10} CFU / day	**12 weeks**<br>***8,4 x 10^{11} CFU***	↓ BMI.<br>↓ Body fat mass.<br>↓ LDL.
	Goebel *et al.* (2012)	100 Obese adolescents with metabolic syndrome	*Lactobacillus salivarius* Ls33	1 x 10^{10} de CFU / day	**12 weeks**<br>***8,4 x 10^{11} CFU***	↔ Anthropometric measures.<br>↔ Cholesterol, LDL and HLD.
	Jung *et al.* (2013)	62 Obese patients	*Lactobacillus gasseri* BNR17	6 × 10^{10} CFU / day	**12 weeks**<br>***5 x 10^{11} CFU***	↓ BMI.<br>↓ Hip circumference.
	Sabico *et al.* (2017)	78 Patients with type 2 diabetes mellitus	*Formula Multispecies*[ii]	5 × 10^{9} CFU / day	**12 weeks**<br>***4,2 x 10^{11} CFU***	↓ Abdominal adiposity
	Ejtahed *et al.* (2012)	60 Patients with type 2 diabetes mellitus	*Lactobacillus acidophilus* La5 and *Bifidobacterium lactis* Bb12	4 x 10^{9} de CFU / day	**6 weeks**<br>***1,7 x 10^{11} CFU***	↓ Glucose.<br>↔ Insulin level.
	Nabavi *et al.* (2015)	72 patients with NAFLD	*Lactobacillus acidophilus* La5 and *Bifidobacterium lactis* Bb12	2,5 × 10^{9} CFU / day	**8 weeks**<br>***1,4 x 10^{11} CFU***	↓ Weight and Body mass.<br>↔ Waist circumference.
	Ivey *et al.* (2014)	156 Overweight men and women	*Lactobacillus acidophilus* La5 and *Bifidobacterium animalis subsp lactis* Bb12	3 × 10^{9} CFU / day	**6 weeks**<br>***1,3 x 10^{11} CFU***	↑ Glucose.
	Gomes *et al.* (2017)	43 Obese women	*Lactobacillus acidophilus* LA-14, *Lactobacillus casei* LC-11, *Lactococcus lactis* LL-23, *Bifidobacterium bifidum* BB-06, and *Bifidobacterium lactis* BL-4	2 x 10^{10} de CFU / day	**8 weeks**<br>***1,1 x 10^{11} CFU***	↓ Plasma polyunsaturated fatty acids.<br>↔ BMI and weight.<br>↓ Waist circumference.
	Chung *et al.* (2016)	40 Obese patients	*Lactobacillus reuteri* JBD301	1 x 10^{9} de CFU / day	**12 weeks**<br>***8,4 x 10^{10} CFU***	↓ BMI.

Koutnikova *et al.* [43]	Barreto *et al.* (2014)	24 Patients with Metabolic syndrome	*Lactobacillus plantarum* Lp 115	1 x 10^9 de CFU / day	**12 weeks** ***8,4 x 10^{10} CFU***	↓ Cholesterol.<br>↓ Glucose.<br>↔ Insulin level.<br>↔ HDL and LDL
	Mohamadshahi *et al.* (2014)	44 Patients with type 2 diabetes mellitus	*Lactobacillus acidophilus* La5 and *Bifidobacterium lactis* Bb12	1,1 × 10^9 CFU / day	**8 weeks** ***6 x 10^{10} CFU***	↔ Anthropometric measures.
	Madjd *et al.* (2016)	89 Obese patients	*Lactobacillus acidophilus* La5 and *Bifidobacterium lactis* Bb12	4 × 10^9 CFU / day	**12 weeks** ***1,7 x 10^9 CFU***	↓ Cholesterol and LDL.
Tenorio *et al.* [13]	Tripolt *et al.* (2013)	28 Subjects with MetS	*Lactobacillus casei* Shirota	1,95 x 10^{10} cells/day	**12 weeks** ***1,6 x 10^{12} cells***	↔ BMI and glucose levels.
	Stadlbauer *et al.* (2015)	28 Patients with MetS	*Lactobacillus casei* Shirota	1,95 x 10^{10} CFU/day	**12 weeks** ***1,6 x 10^{12} CFU***	↔ BMI, waist circumference and TAG.
	Leber *et al.* (2012)	28 Adult patients with MetS	*Lactobacillus casei* Shirota	1,95 x 10^8 CFU/day	**12 weeks** ***1,6 x 10^{10} CFU***	↔ BMI, waist circumference, TAG and glucose levels.
	Szulinska *et al.* (2018)	81 Obese Caucasian post-menopausal women	*Formula Multispecies*[ii]	Low dose 2,5 × 10^9 CFU/day<br>High dose 1 × 10^{10} CFU/day	**12 weeks** ***2,1 x 10^{11} CFU***<br>***8,4 x 10^{11} CFU***	↔ BMI
Caimari *et al.* [37]		40 male Wistar rats induced to MetS	Heat-killed *Bifidobacterium animalis* subsp. *lactis* CECT 8145	10^{10} CFU/day	**12 weeks** ***8,4 x 10^{11} CFU***	↓ energy intake, LDL and VLDL.<br>↑ insulin sensitivity.
Sanz *et al.* [42]	Yadav *et al.* (2008)	Male Wistar rats	*Lactobacillus acidophilus* NCDC14 and *Lactobacillus casei* NCDC19	7,3 x 10^9 CFU/g	**4 weeks** ***2 x 10^{11} CFU***	↓ Insulin secretion.
	Ivanovic *et al.* (2015)	C57BL/6 mice obesity induce by HFD	*Lactobacillus rhamnosus* LA68 and *Lactobacillus plantarum* WCFS1	2 × 10^9 CFU four times per week	**12 weeks** **1,68 x 10^{11} CFU**	↓ Body weight, cholesterol levels.<br>↓ TAG and LDL (WCFS1).

	Ma *et al.* (2008)	Male C57BL / 6J mice with diabetes-induced	VSL#3*	5 × 10^9 CFU/day	**4 weeks** ***1,4 x 10^{11} CFU***	↓ Insulin resistance.
Sanz *et al.* [42]	Laitinen *et al.* (2008)	Women from first trimester of pregnancy	*Lactobacillus rhamnosus* GG and *Bifidobacterium lactis* Bb12	1 x 10^9 CFU/day each strain	**12 weeks** ***8,4, x 10^{10} CFU***	↓ Blood glucose.
	Yadav *et al.* (2007)	Male Wistar rats with diabetes-induced	*Lactobacillus acidophilus* NCDC14 and *Lactobacillus casei* NCDC19	1 x 10^8 CFU/g	**8 weeks** ***5,6 x 10^9 CFU***	↓ Plasma glucose, total cholesterol, TAG, LDL, VLDL and liver glycogen levels. ↓Insulin levels.
	Sato *et al.* (2008)	Male Sprague-Dawley rats	*Lactobacillus gasseri* SBT2055	6 x 10^7 CFU/g	**4 weeks** ***1,7 x 10^9 CFU***	↓ Adipocyte size. ↑ Number of small adipocytes. ↓ Leptin
	Martin *et al.* (2008)	Female *germ-free* mice C3H colonized with human baby microbiota	*Lactobacillus paracasei* NCC2461 and *Lactobacillus rhamnosus* NCC4007	1 x 10^8 CFU/g	**2 weeks** ***1,4 x 10^9 CFU/g***	↑ TAG levels. ↓ VLDL and LDL levels.
Li *et al.* [38]		40 Male Wistar rats with type 2 diabetes and obesity induce by HFD	*Lactobacillus plantarum* NCU116	10^9 CFU/mL	**5 weeks** **3,5 x 10^{10} CFU**	↑ SCFA, insulin, cholesterol levels and TAG. ↓ HDL.
Cano *et al.* [39]		Adult male wild-type C57BL-6 mice, obesity induce by HFD	*Bifidobacterium pseudocatenulatum* CECT 7765	5 × 10^8 CFU/day	**7 weeks** **2,45 x 10^{10} CFU**	↓ Body weight gain and food intake, cholesterol, TAG, glucose, insulin and leptin levels.
Eaimworawuthikul *et al.* [40]		48 male Wistar rats, obesity induce by HFD	*Lactobacillus paracasei* HII01	1 × 10^8 CFU/day	**12 weeks** ***8,4 x 10^9 CFU***	↓ Body weight, LDL, insulin and cholesterol levels.

***VSL#3** (*Streptococcus thermophilus* **DSM24731**, *Bifidobacterium breve* **DSM24732**, *Bifidobacterium longum* **DSM24736**, *Bifidobacterium infantis* **DSM24737**, *Lactobacillus acidophilus* **DSM24735**, *Lactobacillus plantarum* **DSM24730**, *Lactobacillus paracasei* **DSM24733** and *Lactobacillus delbrueckii* subsp. *bulgaricus* **DSM24734**);

Formula multispecies[ii]: *Bifidobacterium bifidum* W23, *Bifidobacterium lactis* W51, *Bifidobacterium lactis* W52, *Lactobacillus acidophilus* W37, *Lactobacillus brevis* W63, *Lactobacillus casei* W56, *Lactobacillus salivarius* W24, *Lactococcus lactis* W19, and *Lactococcus lactis* W58

GLP-1: Glucagon-like peptide-1; **BMI**: body mass index; **aGLP-1**: active Glucagon-like peptide-1; **TAG**: triglycerides; **ALT**: liver toxicity biomarker alanine transaminase; **CFU**: colony-forming unit; **NAFLD**: nonalcoholic fatty liver disease; **LDL**: low-density lipoprotein; **HDL**: high-density lipoprotein; **VLDL**: very low-density lipoprotein

0
